# Can ‘Hebb’ Be Distracted? Testing the Susceptibility of Sequence Learning to Auditory Distraction

**DOI:** 10.5334/joc.8

**Published:** 2018-01-10

**Authors:** François Vachon, Alexandre Marois, Michaël Lévesque-Dion, Maxime Legendre, Jean Saint-Aubin

**Affiliations:** 1 École de psychologie, Université Laval, Québec, CA; 2 Department of Building, Energy and Environmental Engineering, University of Gävle, Gävle, SE; 3 École de psychologie, Université de Moncton, Moncton, CA

**Keywords:** Hebb repetition effect, Sequence learning, Auditory distraction, Serial memory, Irrelevant Sound, Word learning

## Abstract

Sequence learning plays a key role in many daily activities such as language and skills acquisition. The present study sought to assess the nature of the Hebb repetition effect—the enhanced serial recall for a repeated sequence of items compared to random sequences—by examining the vulnerability of this classical sequence-learning phenomenon to auditory distraction. Sound can cause unwanted distraction by either interfering specifically with the processes involved in the focal task (interference-by-process), or by diverting attention away from a focal task (attentional capture). Participants were asked to perform visual serial recall, in which one to-be-remembered sequence was repeated every four trials, while ignoring irrelevant sound. Whereas both changing-state (Experiment 1) and deviant sounds (Experiment 2) disrupted recall performance compared to steady-state sounds, performance for the repeated sequence increased across repetitions at the same rate regardless of the sound condition. Such findings suggest that Hebbian sequence learning is impervious to environmental interference, which provides further evidence that the Hebb repetition effect is an analogue of word-form learning.

The mechanisms underlying the short-term retention and reproduction of serial order information have long been considered a core component of human cognition (e.g., Lashley, 1951). The learning and subsequent use of sequences of information or actions underpin most if not all skilled behaviors (see Marshuetz, 2005). One paradigm developed to understand the processes that underlie sequence learning is the Hebb repetition paradigm (Hebb, 1961). This paradigm is a variation of an immediate serial recall task—requiring participants to remember short sequences of items (e.g., digits) in their order of presentation—in which a particular sequence is repeated at regular intervals, unbeknownst to participants. Typically, recall improves steadily with repetitions of that sequence compared with random sequences (e.g., Couture & Tremblay, 2006; Cummings, Page, & Norris, 2003; Horton, May, & Smyth, 2008; McKelvie, 1987; Oberauer, Jones, & Lewandowsky, 2015). This form of long-term sequence learning is particularly interesting as the Hebb repetition effect is considered as a laboratory analogue of language learning (e.g., Guerrette, Guérard, & Saint-Aubin, 2017; Hitch, Flude, & Burgess, 2009; Mosse & Jarrold, 2008; Page, Cumming, Norris, Hitch, & McNeil, 2006; Page, Cumming, Norris, McNeil, & Hitch, 2013; Page & Norris, 2009b; Saint-Aubin & Guérard, in press; Saint-Aubin, Guérard, Fiset, & Losier, 2015; Szmalec, Duyck, Vandierendonck, Mata, & Page, 2009). In fact, Hebbian sequence learning and language learning appear to be very similar as both forms of learning take place through multiple presentations of verbal sequences and involve the reproduction of sequences of verbal information (e.g., phonemes, syllables) in the same order as such information was perceived. Consistent with that idea, Hebbian sequence learning is impaired in dyslexia, a language-based learning disability (e.g., Szmalec, Loncke, Page, & Duyck, 2011), and can predict reading abilities (e.g., Bogaerts, Szmalec, De Maeyer, Page, & Duyck, 2016).

To supplement the intuition that Hebbian sequence learning and language acquisition are underpinned by common mechanisms, Page and Norris (2009b) listed the properties that the Hebb effect should manifest to be considered as a valid analogue of the sequence-learning component of word-form learning. Current empirical evidence suggests that the Hebb effect does exhibit these properties: i) Hebbian sequence learning is observed independently of the spacing of the repetitions (Page et al., 2013); ii) multiple repeated lists can be learned concurrently (Page et al., 2013; Saint-Aubin & Guérard, in press; Saint-Aubin et al., 2015); the memory trace engendered by the Hebb effect can endure (Page et al., 2013; Szmalec, Page, & Duyck, 2012); iv) Hebbian sequence learning takes place rapidly, requiring only a few repetitions to emerge (e.g., Melton, 1963); v) young children are capable of Hebbian sequence learning (Mosse & Jarrold, 2008); vi) it is possible to learn only portions of repeated lists (Szmalec et al., 2009); and vii) Hebbian sequence learning does not rely on overt language production (e.g., Guerrette et al., 2017; Kalm & Norris, 2016; Oberauer & Meyer, 2009). Hitherto, the accumulating evidence for a functional similarity between word-form learning and Hebbian sequence learning is compelling.

Yet, one important difference between word-form learning and typical Hebb repetition studies has never been addressed: the environmental context in which learning takes place. The Hebb repetition effect is usually observed in controlled laboratory settings largely devoid of potentially distracting interference. However, language acquisition rarely occurs in distraction-free environments. In spite of the ubiquity of distraction in real life settings, particularly in the auditory domain, language can still be learned. This resistance of language acquisition to auditory distraction has been established empirically. There is evidence that the mere presence of extraneous sound—especially speech—can have a negative impact on performance in language-related tasks (e.g., Halin, Marsh, Haga, Holmgren, & Sörqvist, 2014; Klatte, Meis, Sukowski, & Schick, 2007; Sörqvist, Nöstl, & Halin, 2012) without impeding the learning of novel word forms (e.g., Dixon & Salley, 2007). Accordingly, to be a true experimental analogue of word-form learning, the Hebb repetition effect should be resistant to auditory distraction. The present study addressed this issue by testing the vulnerability of Hebbian sequence learning to the presence of irrelevant sound.

Experimental research on the disruptive impact of extraneous sound on cognitive functioning suggests that auditory distraction comes principally in two functionally distinct forms. According to the duplex-mechanism account of auditory distraction (Hughes, 2014; Hughes, Vachon, & Jones, 2005, 2007), irrelevant sound can cause unwanted distraction either by interfering specifically with the processes involved in the ongoing mental activity (interference-by-process) or by diverting attention away from the task at hand (attentional capture). Interference-by-process can be viewed as a form of competition-for-action occurring when the results of obligatory sound processing are similar to those of the focal task. The empirical manifestation par excellence of the interference-by-process mechanism is the *changing-state effect*: the hindering of immediate serial memory by the presence of to-be-ignored sound that is changing acoustically (e.g., Beaman & Jones, 1997; Jones & Macken, 1993; Jones, Madden, & Miles, 1992). The changing-state effect refers to the observation that a sequence of irrelevant changing sounds (e.g., ‘AFBLM…’) presented during the encoding or rehearsal of a serial recall list is far more disruptive than a steady-state sound (‘AAAAA…’). Because the changing-state effect is only obtained when serial rehearsal is a key aspect of the focal task, as in serial recall (e.g., Beaman & Jones, 1997; Hughes et al., 2007), the phenomenon was often imputed to a conflict between two processes involving the maintenance of the order of events (e.g., Hughes, Tremblay, & Jones, 2005; Jones & Macken, 1993; Jones & Tremblay, 2000). More specifically, the obligatory perception of changes in a changing-state auditory sequence yields information about order—that is impoverished or absent in steady-state sound—which competes for, and hence compromises, the deliberate process of rehearsing the to-be-remembered items in serial order.

The second form of auditory distraction within the duplex-mechanism account is attention capture, which refers to a transient exogenous orienting of the attentional focus away from a prevailing mental activity. Attention can be involuntarily diverted because of the specific characteristics of the sound, such as a sound that is significant personally (e.g., one’s own name; Röer, Bell & Buchner, 2013) or particularly arousing (e.g., a taboo word; Röer, Körner, Buchner, & Bell, 2017), or because of the context in which the sound occurs. This latter form of attentional orienting is reflected in the *deviation effect*, that is, the disruption of focal task performance by the rare and unexpected occurrence of a sound that deviates from the context in which it is embedded (e.g., Hughes et al., 2005, 2007; Lange, 2005; Marsh, Röer, Bell, & Buchner, 2014; Parmentier, 2008; Sörqvist, 2010; Schröger & Wolff, 1998; Vachon, Labonté, & Marsh, 2017). For instance, the insertion on a relatively small number of trials of a word spoken in a female voice in an irrelevant sequence of male-spoken words tends to capture attention, momentarily interrupting ongoing action, hence impairing performance (e.g., Hughes, Hurlstone, Marsh, Vachon, & Jones, 2013; Hughes et al., 2007). Contrary to the changing-state effect, the disruptive impact of a deviant sound (or auditory oddball) is not restricted to serial recall and has been observed in a wide range of tasks. In fact, the deviation effect takes place regardless of the particular processing involved in the focal task (Vachon et al., 2017).

With the aim of testing the view that Hebbian sequence learning is an appropriate experimental analogue of language learning, the present study examined the sensitivity of the Hebb repetition effect to both interference-by-process and attention-capture forms of auditory distraction. For the first time, to our knowledge, the Hebb effect was studied under conditions of irrelevant sound. The impact of the changing-state effect was tested in Experiment 1 whereas the deviation effect was investigated in Experiment 2. Given that these two irrelevant-sound phenomena have already been established in a serial recall context, their integration to the Hebb repetition paradigm was therefore straightforward. The distractive (changing-state or deviant) and control conditions (steady-state or without deviant) were contrasted in a between-subjects design to avoid the potential awareness of the repetition in one sound condition contaminating the other sound condition.

## Experiment 1

The aim of Experiment 1 was to test the sensitivity of the Hebb repetition effect to the changing-state effect. To do so, participants performed the typical Hebb procedure (i.e. a visual-verbal serial recall task in which one to-be-remembered sequence was repeated every four trials) in the presence of either steady-state or changing-state irrelevant sound.

### Method

**Participants.** Fifty adults (26 women; mean age: 26.2 years, range: 20–58) were recruited on the campus of Université Laval. All participants reported normal or corrected-to-normal vision and normal hearing. They received a small honorarium for their participation. Half of them were randomly assigned to the steady-state (SS) condition while the other half performed the changing-state (CS) condition.

**Materials.** The experiment was controlled by a PC computer using E-Prime 2.0 Professional (Psychology Software Tools). To-be-remembered visual stimuli were presented on a computer screen located at approximately 60 cm from the participant while to-be-ignored auditory stimuli were presented through Sony Professional headphones at approximately 65 dB(A).

Participants performed a visual-verbal immediate serial recall task. A total of 39 visual sequences were randomly generated. Each sequence was composed of eight digits taken without replacement from the digit set 2–9 and arranged in a quasirandom order, with the constraint that successive digits were not adjacent integers. Each item was approximately 2.5 cm in height and presented sequentially in Times New Roman font at the center of the screen. Each digit was presented for 350 ms, and the interstimulus interval (offset to onset) was 400 ms. One visual sequence was randomly selected for each participant to be used as the repeated (Hebb) sequence while the remaining 38 sequences were assigned to non-repeated (or random) trials.

**Figure 1:**
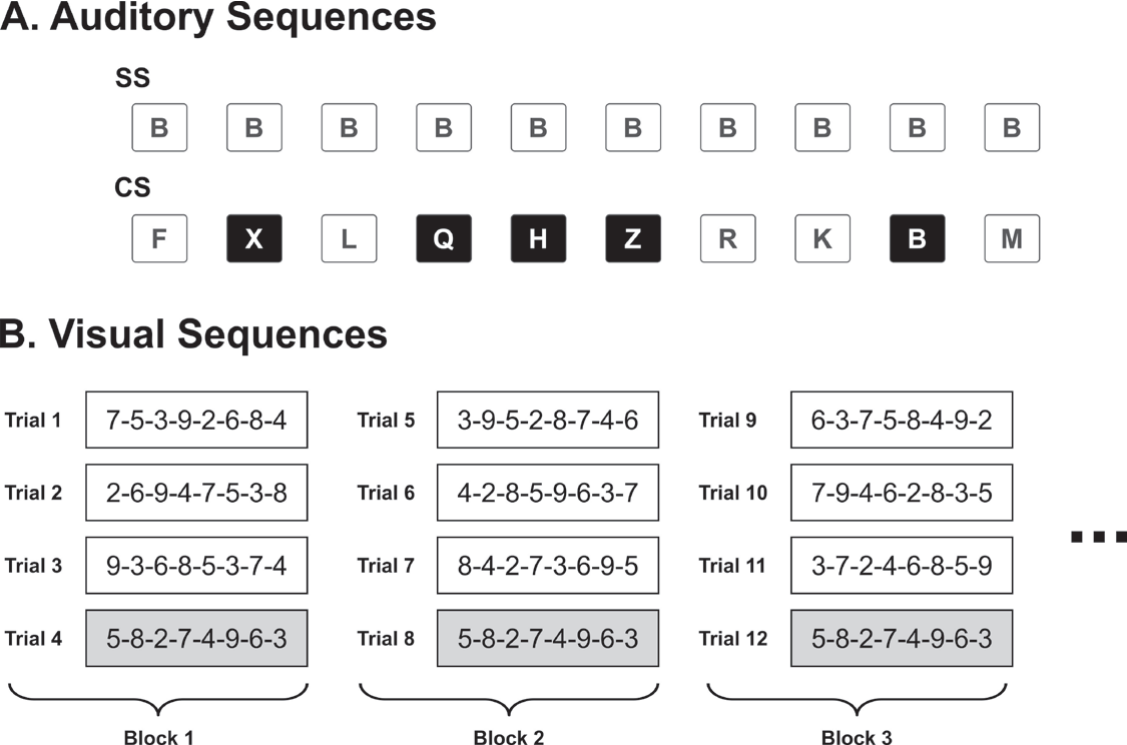
Illustration of the stimuli employed in Experiment 1. **A**: Schematic representation of the to-be-ignored sequence of spoken letters presented in each sound condition: steady state (SS) vs. changing state (CS). A white box represents a letter spoken in a female voice while a black box represents a letter spoken in a male voice. **B**: Example of the to-be-remembered sequence of visual digits presented in the first 12 trials (i.e. first three blocks of trials). A white box represents a random sequence while a gray box represents the repeated sequence. In this experiment, the repeated sequence was always presented in the fourth (i.e. last) trial of each block.

For the irrelevant auditory sequences, four sets of the the letters *B-F-H-K-L-M-Q-R-X-Z* were recorded in four different voices (2 male voices and 2 female voices) at an approximately even pitch and edited using SoundForge software (Sony) so that each lasted 250 ms. Each auditory sequence comprised 10 spoken letters separated from each other by an interstimulus interval of 350 ms. In the SS condition, a single sequence consisted of 10 repetitions of the letter ‘B’ spoken in the same female voice was used in every trial (see Figure 1A). In the CS condition, the auditory sequence was composed of all 10 letters presented in a different random order and in different voices for each trial. Each letter was spoken in a different, randomly selected voice so that acoustic variability in CS sequences was realized via both changes of item and changes of voice. The onset of the first letter-sound occurred 125 ms before the onset of the first visual digit, and the offset of the final letter-sound preceded the offset of the final visual digit by 75 ms.

**Design and procedure.** Participants were assigned either to the SS or to the CS condition. The experimental session involved 48 trials divided into 12 blocks. Each block consisted of three unique, random sequences and one repeated sequence presented on the fourth trial of the block (see Figure 1B). The exact same repeated sequence and random sequences order were assigned to a single participant in each group. This means that the only difference between the two groups was the type of auditory sequences presented. These manipulations yielded a 2 × 2 × 12 mixed design with Sound condition (SS and CS) as the between-subjects factor and Repetition (repeated and random) and Block of trials (1 to 12) as the within-subject factors.

Participants were tested individually in a sound-attenuated room. They read standard instructions informing them to recall the order of presentation of the eight digits and asking them to ignore any sounds presented over the headphones. They were not told about the presence of the repeated to-be-remembered sequence. Participants had to complete two practice trials with a random visual sequence before performing the experimental trials. Four hundred milliseconds following the presentation of the last digit, all digits reappeared horizontally in canonical order. Participants had to click on the digits using the mouse in the order in which they had been presented. Each item turned green once selected. No omissions were allowed. Once participants had recalled the whole sequence, they pressed the spacebar to begin the next trial. The experiment lasted approximately 25 min.

### Results and Discussion

The raw data were scored according to the strict serial recall criterion: To be recorded as correct, an item had to be recalled in its original presentation position. The percentage of correct recall was first analyzed according to the 2 (Sound condition) × 2 (Repetition) × 12 (Block of trials) mixed design in order to assess the impact of sound on performance. We then computed the gradient of improvement to examine whether sound affected the learning rate. All dependent variables were analyzed using mixed ANOVAs. Therefore, the Greenhouse-Geisser procedure was applied to every within-subject effect for which the sphericity assumption was violated. The critical α was fixed at .05 for all analyses.

**Figure 2:**
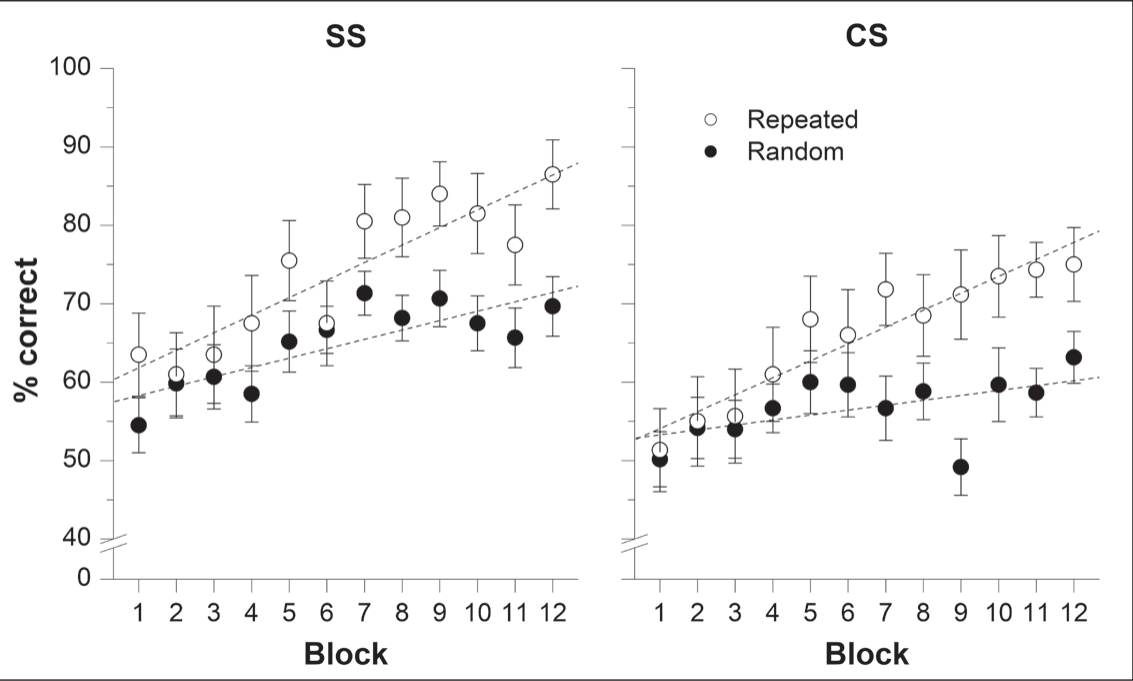
Results from Experiment 1: Percentage of correct recall as a function of Block of trials for repeated (one trial per block) and random sequences (three trials per block) in the steady-state (SS) and changing-state (CS) sound conditions. Error bars represent the standard error of the mean. Regression lines were computed separately for repeated and random trials.

**Recall performance.** Figure 2 shows the percentage of items correctly recalled across the 12 blocks of trials for repeated and random trials in each sound condition. The mixed ANOVA revealed a main effect of Sound condition, *F*(1, 48) = 4.77, *p* = .034, η^2^_p_ = .090, indicating that serial recall was poorer in CS (*M* = 61.3%) than in SS trials (*M* = 69.5%), a replication of the CS effect. The main effect of Repetition was significant, *F*(1, 48) = 33.19, *p* < .001, η^2^_p_ = .409, so was the main effect of Block of trials, *F*(11, 528) = 9.82, *p* < .001, η^2^_p_ = .170. Importantly, the interaction between Repetition and Block of trials was significant, *F*(8.305, 398.635) = 2.48, *p* = .011, η^2^_p_ = .049, indicative of a Hebb learning effect, i.e. a greater improvement of performance over blocks for repeated than random sequences. None of the interactions involving Sound condition reached significance (*F*s < 1). Notably, the non-significant three-way interaction (*F*(8.305, 398.635) = 0.54, *p* = .835, η^2^_p_ = .011) suggested that there was no difference between the Hebb repetition effect found in the SS and the CS conditions. A Bayes factor analysis showed that the Bayes factor for this three-way interaction term is 8.43 × 10^9^, which yields very strong support for the null hypothesis, *p*_BIC_(H_0_|D) > .999 (see Masson, 2011).

**Gradient of improvement.** Gradients of improvement were computed to examine whether performance increased over repetitions. For both sound conditions, the percentage of correct responses were calculated for each participant by collapsing performance across serial positions for each repeated and random trial. Performance was then averaged across the three random trials of each block. Linear regressions were then computed separately for the repeated and random trials on the mean proportion of correct recall in each block, averaged across participants. Using such a calculation provided us with individual slopes that reflected improvement over repetitions. The mean improvement observed for repeated and random trials are provided in Table 1 for both SS and CS sound conditions. A 2 × 2 mixed ANOVA was performed on these data with Sound condition (SS and CS) as the between-subjects factor and Repetition (repeated and random) as the within-subject factor. The significant main effect of Repetition, *F*(1, 48) = 13.92, *p* = .001, η^2^_p_ = .225, indicated that the gradient of improvement was higher for the repeated sequence than for the random sequences. This result confirmed the presence of a Hebb repetition effect. The main effect of Sound condition was not significant, *F*(1, 48) = 0.75, *p* = .390, η^2^_p_ = .015. More importantly, the interaction between Sound condition and Repetition also failed to reach significance, *F*(1, 48) = 0.56, *p* = .480, η^2^_p_ = .010, suggesting that the improvement advantage for the repeated sequence was not affected by the type of auditory stimulation. This conclusion is supported by a Bayes factors analysis yielding a Bayes factor of 5.43, which provided positive support for the null hypothesis, *p*_BIC_(H_0_|D) = .845. To scrutinize this absence of effect of CS sound on the Hebb repetition effect, we contrasted the gradients of improvement for the repeated sequence across the two sound conditions. The ANOVA revealed no significant difference, *F*(1, 48) = 0.16, *p* = .901, η^2^_p_ < .001, while a Bayes factor of 7.02 yielded positive support for the null hypothesis, *p*_BIC_(H_0_|D) = .875.

**Table 1:** Mean gradient of improvement (+*SE*), in percentage, for repeated and random trials in the steady-state (SS) and changing-state (CS) sound conditions of Experiment 1.

		Repetition	
		Repeated	Random

Sound condition	SS	2.236 (0.458)	1.154 (0.265)
	CS	2.159 (0.418)	0.626 (0.244)

Overall, Experiment 1 replicated the classical Hebb repetition and changing-state effects but failed to reveal an impact of this form of auditory distraction on the Hebb repetition effect. Indeed, despite serial recall performance being lower in the presence of changing-state than steady-state irrelevant sound, the learning rate of the repeated sequence remained unaffected by the presence of changing-state sound. Such findings suggest that the Hebb repetition effect is impervious to auditory distraction, at least when such distraction is driven by interference-by-process.

## Experiment 2A

**Figure 3:**
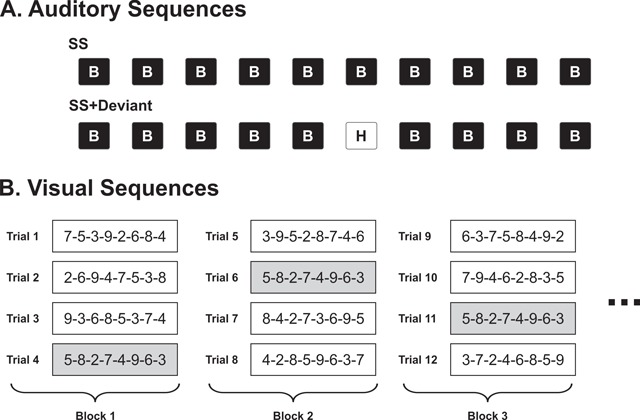
Illustration of the stimuli employed in Experiment 2A. **A**: Schematic representation of the to-be-ignored sequence of spoken letters presented in each sound condition: steady state (SS) vs. SS+Deviant. A white box represents a letter spoken in a female voice while a black box represents a letter spoken in a male voice. In this example, a deviant item is presented in the sixth position. **B**: Example of the to-be-remembered sequence of visual digits presented in the first 12 trials (i.e. first three blocks of trials). A white box represents a random sequence while a gray box represents the repeated sequence. In this experiment, the repeated sequence was presented randomly in any of the four trials of each block, with the constraint that there was at least one random trial (here, Trial 5) interleaved between repeated trials (here, Trials 4 and 6).

This experiment was designed to examine the impact of attentional capture by deviant sounds on Hebbian sequence learning. To do so, participants carried out the same Hebb procedure as in Experiment 1, but this time in the presence of either steady-state irrelevant sound or steady-state sound sequences in which a deviant item was embedded. In order to avoid presenting too many deviant events throughout the experimental session, hence weakening their potency, deviant sounds were exclusively presented on repeated trials (i.e. in 25% of trials). Given that the attention-capture power of a deviant sound relies on its unexpectedness (e.g., Hughes et al., 2013; Marsh et al., 2014; Sussman, Winkler, & Schröger, 2003; Vachon, Hughes, & Jones, 2012), repeated trials could then not take place in a predictable fashion like in Experiment 1 (i.e. in every four trials). Therefore, in order to make sure the occurrence of the deviant could not be predicted, the repeated trial of each of the 12 blocks of trials was presented randomly in any of the four trials of a block (see Figure 3B). This is a departure from the classical Hebb procedure (see also St-Louis, Hughes, Saint-Aubin, & Tremblay, 2017) as the repeated sequence is always presented at a constant rhythm within a single experimental session (e.g., in every four trials; see, e.g., Couture & Tremblay, 2006; Cumming et al., 2003; Hebb, 1961; Horton et al., 2008; Oberauer et al., 2015; Page et al., 2013; Szmalec et al., 2009). Such a variation of the paradigm is not expected to exert an influence on the Hebb repetition effect, however, as the phenomenon is deemed not critically dependent on the spacing between sequence repetitions (see Page & Norris, 2009b). Given the repeated sequence can sometimes become consciously recognized over repetitions (e.g., McKelvie, 1987), the occurrence of a deviant sound that always accompanied the repeated sequence could then become expected. To avoid this, the deviant unpredictability was also promoted by varying both its position within the auditory sequence and its acoustical features across sequences. There is evidence that the unpredictable timing of a to-be-expected auditory deviation (it was impossible to know beforehand when it would take place in the sequence) is sufficient to trigger an attentional response (e.g., Marois, Labonté, Parent, & Vachon, in press), hence disrupting the ongoing cognitive activity.

Because a deviant sound systematically accompanied every presentation of the repeated sequence in the SS+deviant condition, an improvement of performance over repetitions would mimic a pattern of habituation of the attentional response to the deviant. Accordingly, a control experiment (Experiment 2B) was run to rule out this alternative hypothesis.

### Method

The method was identical to that of Experiment 1, except as noted below.

**Participants.** Forty adults (24 women; mean age: 26.8 years, range: 19–60) took part in this experiment. None of them participated in Experiment 1. They were randomly assigned to either the SS or the SS+deviant condition.

**Materials and procedure.** Figure 3A illustrates the two type of auditory sequences employed in this experiment. In the SS condition, the auditory sequence used in every trials consisted in 10 repetitions of the letter ‘B’ spoken in a male voice. In the SS+deviant, this SS sequence was presented in random trials only. The auditory sequence accompanying the repeated to-be-remembered sequence was identical to the SS sequence except that one of the letters occurring randomly between the fourth and seventh position was replaced by a (deviant) letter taken from the set *F-H-K-L-M-Q-R-X-Z* and spoken in a female voice. Hence, deviant sounds were restricted to repeated trials. As in the previous experiment, the experimental session comprised 12 blocks of four trials, each block consisting of one repeated trial and three random trials. However, to avoid the occurrence of a deviant to be predictable, the repeated trial was randomly assigned to one of the four trials of each block, with the constraint that there was at least one random trial interleaved between repeated trials (see Figure 3B).

### Results and Discussion

**Figure 4:**
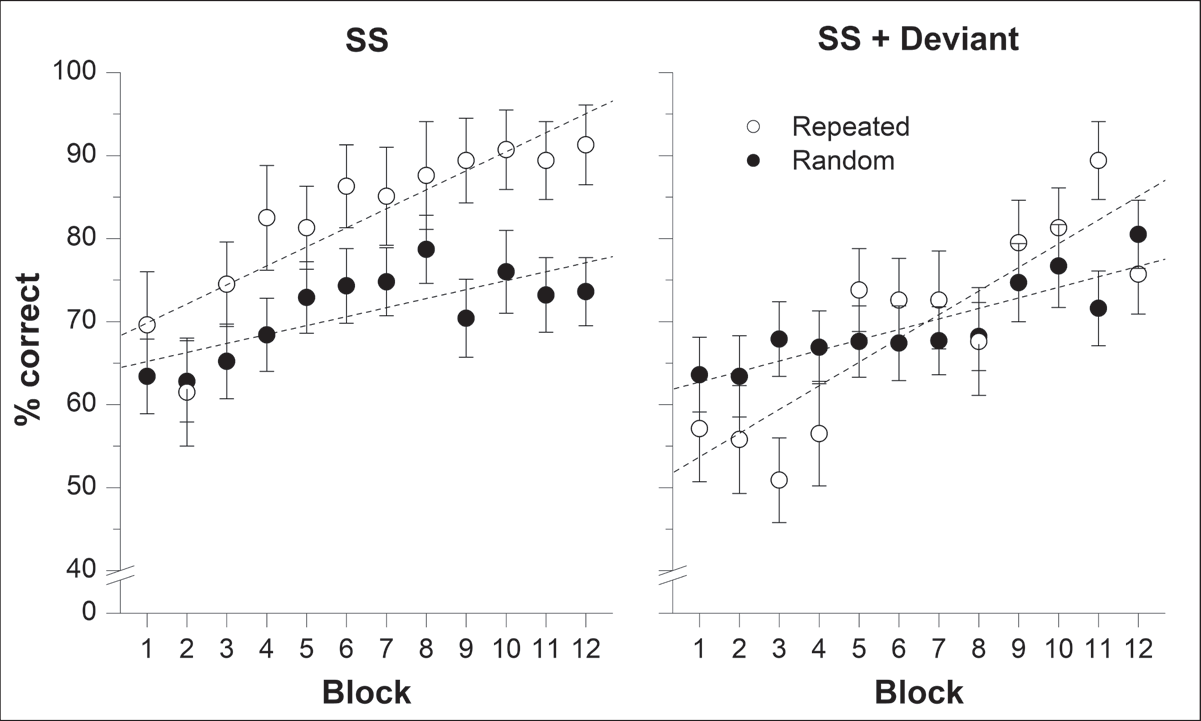
Results from Experiment 2A: Percentage of correct recall as a function of Block of trials for repeated (one trial per block) and random sequences (three trials per block) in the steady-state (SS) and SS+deviant sound conditions. Error bars represent the standard error of the mean. Regression lines were computed separately for repeated and random trials.

**Recall performance.** Figure 4 shows the percentage of items correctly recalled across the 12 blocks of trials for repeated and random trials in each sound condition. The mixed ANOVA carried out on these data revealed significant main effect of Repetition, *F*(1, 38) = 7.54, *p* = .009, η^2^_p_ = .166, and of Block of trials, *F*(11, 418) = 11.74, *p* < .001, η^2^_p_ = .236. Importantly, the interaction between Repetition and Block of trials was significant, *F*(11, 418) = 2.74, *p* = .002, η^2^_p_ = .067, revealing the presence of a Hebb repetition effect. Whereas the main effect of Sound condition did not reach significance, *F*(1, 38) = 2.95, *p* = .094, η^2^_p_ = .072, the interaction between Sound condition and Repetition was significant, *F*(1, 38) = 8.33, *p* = .006, η^2^_p_ = .180. This interaction arose because recall was better in repeated (*M* = 82.42%) than in random trials (*M* = 71.11%) in the SS condition, but not in the SS+deviant condition (repeated: *M* = 69.67% vs. random: *M* = 69.38%). The fact that overall performance was similar in repeated and random trials when a deviant sound was embedded in the irrelevant auditory sequence of repeated trials is indicative of a deviation effect. However, Sound condition did not interact with Block of trials, *F*(11, 418) = 1.24, *p* = .255, η^2^_p_ = .032, nor with both Block of trials and Repetition, *F*(11, 418) = 1.35, *p* = .195, η^2^_p_ = .034. The absence of a significant three-way interaction suggests that the deviation effect had no impact on the expression of the Hebb repetition effect. A Bayes factor analysis of the three-way interaction produced a Bayes factor of 2.18 × 10^9^, yielding very strong support for the null hypothesis, *p*_BIC_(H_0_|D) > .999.

**Gradient of improvement.** The mean improvement observed for repeated and random trials are provided in Table 2 for both SS and SS+deviant sound conditions. A 2 × 2 mixed ANOVA performed on these data showed a significant main effect of Repetition, *F*(1, 38) = 19.06, *p* < .001, η^2^_p_ = .334, confirming the presence of a Hebb repetition effect. The main effect of Sound condition was not significant, *F*(1, 38) = 0.57, *p* = .453, η^2^_p_ = .015. More importantly, the interaction between Sound condition and Repetition was also not significant, *F*(1, 38) = 0.36, *p* = .553, η^2^_p_ = .009, suggesting that the improvement advantage for the repeated sequence was not affected by the presence of an auditory deviation. This conclusion is supported by a Bayes factors analysis yielding a Bayes factor of 5.24, which provided positive support for the null hypothesis, *p*_BIC_(H_0_|D) = .840. To scrutinize this absence of deviation effect on the Hebb repetition effect, we contrasted the gradients of improvement for the repeated sequence across the two sound conditions. The ANOVA revealed no significant difference, *F*(1, 38) = 0.69, *p* = .410, η^2^_p_ = .018, while a Bayes factor of 3.73 yielded positive support for the null hypothesis, *p*_BIC_(H_0_|D) = .789.

**Table 2:** Mean gradient of improvement (+*SE*), in percentage, for repeated and random trials in the steady-state (SS) and SS+deviant sound conditions of Experiment 2A.

		Repetition	
		Repeated	Random

Sound condition	SS	2.306 (0.460)	1.088 (0.335)
	SS+Deviant	2.872 (0.498)	1.266 (0.344)

Overall, the results of Experiment 2A revealed that the deviation effect observed for repeated trials did not influence the learning of that repeated sequence. In fact, although as expected, recall performance was poorer in repeated (deviant) trials than in random (non-deviant) trials in the first blocks of trials, the learning of the repeated sequence was not affected compared to a condition with no auditory deviation (see Figure 4). Such findings suggest that the Hebb repetition effect is resistant to auditory attentional capture.

## Experiment 2B

One could argue that the improvement of serial recall over repetitions of the repeated sequence observed in Experiment 2A was not due to the learning of that sequence but was instead attributable to the habituation to the auditory deviation. There is indeed ample evidence that the attentional response to a deviant sound can habituate—i.e. decrease—over repeated presentations of that deviant (e.g., Gati & Ben-Shakhar, 1990; Cowan, 1995; Marois et al., in press; Sokolov, 1963; Vachon et al., 2012). Despite our efforts to make the occurrence of the deviant as unexpected and hence as potent as possible (e.g., the deviant sound changed from trial to trial; the occurrence of the deviant was unpredictable across trials and within the auditory sequence), it is possible that the mere repeated encounters with a deviant event was enough to reduce the orienting response to such a deviation. To rule out this alternative hypothesis, we ran an exact replication of Experiment 2A, but we replaced the repeated sequence by random sequences. If the improvement of the recall of the repeated sequence found in the previous experiment ensued from the habituation of the attentional response to the deviant sound, the same improvement over blocks should be obtained for deviant trials in Experiment 2B despite the absence of a repeated sequence.

**Figure 5:**
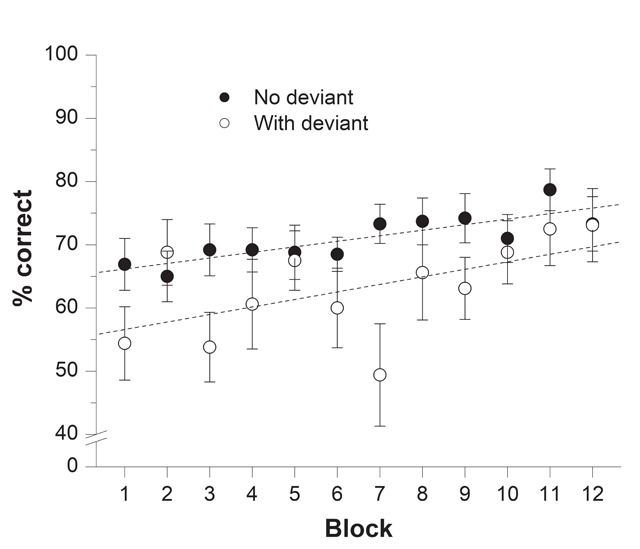
Results from Experiment 2B: Percentage of correct recall as a function of Block of trials for deviant (one trial per block) and non-deviant trials (three trials per block). Error bars represent the standard error of the mean. Regression lines were computed separately for deviant and non-deviant trials.

Twenty new participants (11 women; mean age: 24.9 years, range: 19–34) were recruited to take part in this experiment. The results are presented in Figure 5. The 2 (Deviation: with and without deviant) × 12 (Block of trials) mixed ANOVA carried out on these data showed that performance was poorer in deviant (*M* = 69.5%) than in non-deviant trials (*M* = 76.8%), *F*(1, 19) = 21.08, *p* < .001, η^2^_p_ = .526. Although performance significantly improved over blocks of trials, *F*(11, 209) = 2.27, *p* = .012, η^2^_p_ = .107, it did at a similar rate for both types of trials, *F*(11, 209) = 1.34, *p* = .202, η^2^_p_ = .066. A Bayes factor analysis of the interaction produced a Bayes factor of 4.16 × 10^9^, which provides very strong support for the null hypothesis, *p*_BIC_(H_0_|D) > .999. To further test the habituation hypothesis, we contrasted the gradient of improvement obtained in the deviant trials of Experiment 2B (*M* = 1.194%, *SE* = 0.384) to that observed in the deviant (i.e. repeated) trials of Experiment 2A (*M* = 2.872%, *SE* = 0.498). The one-way ANOVA confirmed the learning rate was faster with the repeated sequence, *F*(1, 38) = 7.12, *p* = .011, η^2^_p_ = .158. A Bayes factor analysis of this effect yielded a Bayes factor of 0.165, providing positive support, this time, against the null hypothesis, *p*_BIC_(H_0_|D) = .142. Such a pattern of results provided clear evidence that the improvement advantage for the repeated sequence of Experiment 2A cannot be explained in terms of habituation to the deviant sound.

## General Discussion

The present study was concerned with one critical property the Hebb repetition effect should exhibit as a valid laboratory analogue of language learning and that has not yet been tested: its resilience to the distractive power of extraneous sound. Two experiments showed that the disruptive impact of changing-state and deviant sounds on serial recall did not influence the rate at which the repeated sequence was learned. These results revealed that Hebbian sequence learning could take place in spite of the presence of interfering (auditory) stimulation that impaired the execution of the focal task. In fact, the learning rate of the repeated sequence remained unaffected regardless of whether cognitive functioning was impeded by interference-by-process or attentional capture. These findings provide further support that Hebbian sequence learning and word-form learning are subtended by common mechanisms.

### Insensitivity to Interference-by-Process

Although the present study was the first, to our knowledge, to embed irrelevant sound within the Hebb repetition paradigm, its distractive impact has been tested in other sequence learning paradigms. In the serial reaction time (SRT) task (Nissen & Bullemer, 1987), participants are presented with a speeded task during which they have to respond to the location of a visual target stimulus appearing at one of several possible locations. The series of locations is structured to follow a regularity that is often repeated over many cycles, without participants’ knowledge (see Schwarb & Schumacher, 2012, for a review). The typical result is a rapid decrease in response times across blocks of the repeated series, indicative of some learning of the sequential regularity (e.g., Curran & Keele, 1993; Jiménez & Vázquez, 2005; Keller & Koch, 2008; Willingham, Nissen, & Bullemer, 1989). Consistent with the results of the present study, Farley, Neath, Allbritton, and Surprenant (2007) showed that the presence of changing-state to-be-ignored sounds impaired performance at the SRT task without affecting the rate of learning. This functional similarity between the Hebb and SRT effects with regard to their resilience to irrelevant sound suggests that common processes underpin sequence learning manifested in the two paradigms (see also Guérard, Saint-Aubin, Boucher, & Tremblay, 2011; Stadler, 1993).

However, in the context of statistical learning, which refers to the ability to detect statistical regularities in the environment, Neath, Guérard, Jalbert, Bireta, and Surprenant (2009) reported a diminished capacity at discriminating already-encountered statistical structure within a stream of shapes from random sequences of the same shapes under conditions of changing-state sound compared with conditions of steady-state sound or quiet. In the light of the present results and those of Farley and colleagues (2007), this demonstration that statistical learning is vulnerable to irrelevant changing sounds appears, at first blush, puzzling as the SRT and Hebb repetition tasks “are all considered to be proxies of statistical learning, because they all involve learning statistical regularities” (Siegelman, Bogaerts, Christiansen, & Frost, 2017, p. 5). However, such discrepancies across these various forms of learning can be accounted for by differences in the way learning was computed in these studies. Whereas sequence learning was assessed through the improvement of performance—in terms of accuracy or response time—over repetitions in the Hebb and SRT paradigms, statistical learning was measured using a familiarity judgment whereby participants indicated which one of two sequence excerpts presented one after the other was the most familiar, only one matching the pattern of the previously-presented sequence. The output of such a familiarity judgement does certainly reflect the action of processes other than the learning of statistical regularities. The engagement of serial processes sensitive to changing-state sound interference such as the serial encoding of the two excerpts and the comparison of their respective sequential representation might have translated into the drop in overall performance observed by Neath and colleagues. A more pure measure of statistical learning would have certainly led to a different pattern of results.

According to the interference-by-process account of the changing-state effect, the automatic extraction of cues pertaining to the order of acoustically changing sounds conflicts with the deliberated maintenance of order of the to-be-remembered stimuli (e.g., Jones & Macken, 1993; Jones & Tremblay, 2000). Therefore, the absence of changing-state distraction on the Hebb repetition effect suggests that the seriation process plays little if any role in Hebbian sequence learning in spite of its importance for short-term retention. Such a conclusion is consistent with studies showing that the Hebb effect remained unaffected under conditions of articulatory suppression (Horton et al., 2008; Page et al., 2006), a manipulation known to severely constrain serial rehearsal. The idea that seriation does not contribute to Hebbian sequence learning is also consistent with the study of Oberauer and Meyer (2009), who showed that the addition of a retention interval before recall—a manipulation designed to promote rehearsal—had no impact on the Hebb repetition effect. Altogether, these findings argue against the view that active rehearsal is the key process underpinning the Hebb repetition effect (e.g., Cunningham, Healy, & Williams, 1984; Kidd & Greenwald, 1988).

None of the theoretical accounts of the Hebb repetition effect makes explicit predictions about the impact of auditory distraction on the learning of the repeated sequence. Yet, within the computational framework of the primacy model of immediate serial recall (Page & Norris, 1998), Page and Norris offered an account of both the Hebb repetition effect (Page & Norris, 2009a) and the changing-state effect (Page & Norris, 2003). According to a revised version of the primacy model (Page & Norris, 2009a), the presentation of a novel to-be-remembered sequence activates several subsequence (or chunk) representations that have not yet been committed (even partially) to any chunk. These chunk representations compete with one another to determine which one will be the best at representing that sequence. The winner of the competitive process is allocated—i.e. becomes engaged—to the sequence, meaning that every sequence in the experiment is associated with a chunk representation on its first occurrence. Such ‘engagement’ (or provisional commitment) is the basis of the learning process: on subsequent presentations of the (repeated) sequence, the previously engaged chunk representation will be better activated. The representation is assumed to become more and more order-selective over repetitions, hence the gradual improvement of the recall of the repeated sequence.

Before proposing this explanation of Hebbian sequence learning, Page and Norris (2003) put forward an account of the changing-state effect within the primacy model that shares some similarities with the interference-by-process view. According to the authors, the presentation of changing-state sound, albeit irrelevant, indicates the presence of ordered information, which prompts the setting up of a chunk representation for—a subsection of—that auditory sequence. To avoid direct interference, this representation of the auditory items needs to be streamed separately from that of the visual items. The establishment of this ‘second’ chunk representation is therefore assumed to divert order-representing resources away from the chunk representation of the concurrently presented to-be-remembered sequence. Changing-state interference ensues from a competition for resources between the chunk representation of the visual list and that of the auditory stream. The consequence of such competition has been quantitatively implemented as a drop of resources necessary to maintain the activation of the engaged chunk representation of the to-be-remembered sequence (see Page & Norris, 2003). Put simply, the presence of changing-state irrelevant sound would tend to weaken the activation of the representation of task-relevant order information.

When applied to the context of the Hebb repetition procedure, this account presumes that the representation of all to-be-remembered sequences—including the repeated sequence—will be less strongly activated under conditions of changing-state sound. With a lower level of activation on the first and subsequent presentations, the representation of the repeated sequence should therefore require more repetitions before becoming fully committed (i.e. completely learned). In other words, Hebbian sequence learning should be slower in the presence of changing-state sound compared with steady-state sound or quiet. This prediction of the primacy model is not supported by the present data, which revealed no influence of irrelevant sound on the Hebb effect. In fact, the model sets the locus of interference of changing-state sound at the same stage where Hebbian sequence learning takes place while the present results clearly showed a dissociation between the ability of accurately recalling serial order and that of learning it (cf. Horton et al., 2008; Page et al., 2006). Modelling of Hebbian sequence learning will need to consider such a dissociation.

### Insensitivity to Attentional Capture

A thorough understanding of the contribution of attentional processes to the Hebb effect has been hampered by a relative dearth of studies. In fact, the only study, to our knowledge, that has directly addressed this issue so far suggested that devoting some attention to the to-be-remembered items is a prerequisite for Hebbian sequence learning to take place. Indeed, Kidd and Greenwald (1988) found no Hebb effect when the repeated sequence was presented (on 10 consecutive trials) in an unattended auditory channel concurrently to a to-be-processed sequence presented in another channel. Towards filling this gap in the literature on the Hebb effect, the present study demonstrated that attention capture by deviant sounds disrupted serial recall without altering the Hebbian sequence learning. Because language acquisition usually takes place in noisy environments where potentially attention-grabbing stimulations are common, it was no surprise to find a reliable Hebb repetition effect in the SS+deviant condition. However, our results also showed that even if attention was momentarily diverted away from the repeated sequence, the rate of learning of that sequence remained unaffected compared with a full-attention condition. Such findings point to at best a limited role for attention in Hebbian sequence learning, which is consistent with a view of implicit sequence learning that considers this form of learning as the result of an automatic associative process running independently of attentional load (e.g., Jiménez & Méndez, 1999; Seger, 1994; Stadler, 1993).

### Temporal Irregularity in the Exposure to the Repeated Sequence

The Hebb repetition effect is typically observed in a context in which the repeated sequence is constantly presented at regular intervals (usually every three or four trials; see, e.g., Couture & Tremblay, 2006; Cumming et al., 2003; Hebb, 1961; Oberauer et al., 2015; Page et al., 2013). In Experiment 2A, we showed that the Hebb effect took place even if the repeated sequence was randomly separated from each other by one to six random sequences. This resilience of Hebbian sequence learning to the irregular presentations of the repeated material (see also St-Louis et al., 2017) provides further support to the view that this form of learning is a valuable laboratory analogue of word-form learning. Indeed, in natural language acquisition, novel word-forms are virtually never repeatedly encountered in a regular or fixed fashion (as in the Hebb repetition paradigm), and such word-forms can nonetheless be learned.

## Conclusion

The acquisition of language is fundamental and the Hebb repetition paradigm is viewed as a key experimental procedure to capture the basics of word-form learning. The present study adds to the accumulating evidence that the Hebb repetition effect is a valid laboratory analogue of language learning by establishing its resilience to sound distractibility. This property of the Hebb repetition effect is critical, as children do not acquire language in a sound-attenuated room. The present set of data poses an interesting challenge for the modelling of Hebbian sequence learning as the capability of maintaining order information over the short term dissociates from the aptitude for the long-term learning of that information.

## Data Accessibility Statement

The data are publicly available at https://doi.org/10.3886/E101179V1.
